# Sitting, standing and moving during work and leisure among male and female office workers of different age: a compositional data analysis

**DOI:** 10.1186/s12889-020-08909-w

**Published:** 2020-06-01

**Authors:** Elin Johansson, Svend Erik Mathiassen, Charlotte Lund Rasmusse, David M. Hallman

**Affiliations:** 1 Centre for Musculoskeletal Research, Department of Occupational Health Sciences and Psychology, University of Gävle, Gävle, Sweden; 2 National Research Centre for the Working Environment, Copenhagen, Denmark; 3 Section of Social Medicine, Department of Public Health, University of Copenhagen, Copenhagen, Denmark

**Keywords:** Accelerometry, Age, CoDA, Gender, Occupational, Physical activity

## Abstract

**Background:**

Gendered patterns of physical activity behaviours may help explaining health inequalities between men and women. However, evidence on such patterns in the working population is sparse. This study aimed at documenting and comparing compositions of sitting, standing and moving at work and during leisure among male and female office workers of different age.

**Methods:**

Sitting (including lying), standing and moving were measured using accelerometry for, on average, four working days in 55 male and 57 female Swedish office workers. Behaviours were described in terms of time spent in four exhaustive categories: sitting in short (< 30 min) and long (≥30 min) bouts, standing, and moving. In a compositional data analysis approach, isometric log-ratios (ilr) were calculated for time sitting relative to non-sitting, time in short relative to long sitting bouts, and time in standing relative to moving. Differences between genders (men vs. women), domains (work vs. leisure), and according to age were examined for each ilr using ANOVA.

**Results:**

At work, time spent sitting in short bouts, sitting in long bouts, standing, and moving was, on average, 29, 43, 21 and 7% among men, and 28, 38, 26 and 7% among women. Corresponding proportions during leisure were 34, 27, 27 and 13% among men and 28, 27, 32 and 13% among women. Men spent more time sitting relative to non-sitting (${\eta}_p^2$ =0.04, *p* = 0.03) than women, and less time standing relative to moving (${\eta}_p^2$ =0.07, *p* = 0.01). At work compared to during leisure, both genders spent more time sitting relative to non-sitting (${\eta}_p^2$ =0.47, *p* < 0.01); within sitting more time was spent in long relative to short sitting bouts (${\eta}_p^2$ =0.26, *p* < 0.01), and within non-sitting, more time was spent standing than moving (${\eta}_p^2$ =0.12, *p* < 0.01). Older workers spent less of their non-sitting time moving than younger workers (${\eta}_p^2$ =0.07, *p* = 0.01).

**Conclusion:**

Male office workers spent more time sitting relative to non-sitting than female workers, and more time moving relative to standing. Both genders were sitting more at work than during leisure. Older workers moved less than younger. These workers could likely benefit from interventions to reduce or break up prolonged sitting time, preferably by moving more.

## Background

A large proportion of the work force world-wide spends a major part of their working days in office settings, being to a large extent sedentary, for instance while doing computer work [1]. Health consequences of sedentary behaviour, defined as any waking behaviour characterized by an energy expenditure ≤1.5 METs while in a sitting, reclining or lying posture [2], have been extensively studied [3–5]. It is well known that sedentariness and physical inactivity negatively affect cardiovascular health and mortality [5, 6]. Some studies suggest that breaking up prolonged sedentary behaviour in shorter periods improves cardio-metabolic health markers compared to spending the same total time in longer periods [7]. However, this needs to be confirmed by more high-quality studies [8].

Some studies have found that men and women within the same occupation may perform different work tasks [9], which will likely affect the occurrence and temporal pattern of sedentary behaviour and physical activities at work. As one example, female call-centre workers have been shown to spend more time in prolonged sedentary behaviour compared with their male colleagues [10]. Men and women may differ in sedentary and physical activity behaviours also outside work (i.e. during leisure time), such as reported among blue-collar workers [11, 12]. These differences could contribute in explaining why women have a higher prevalence of musculoskeletal complaints than men [13, 14]. However, little evidence is available on gendered patterns of sedentary behaviour and physical activities among male and female office workers. Furthermore, sedentary behaviour and physical activities can be expected to change with age [15, 16]. The extent of this change, and whether it differs between women and men [17] has not been addressed for office workers.

Both work and leisure contribute to the overall occurrence and temporal pattern of sedentary behaviour and physical activity of an individual, and leisure time physical activity has the potential to reduce cardiovascular risks associated with sedentary work [5, 18]. However, the extent to which individuals with much sedentary time at work are physically active during leisure is still an open question. A recent paper in the present journal emphasized that documentations of time use in different physical activities during the day(s) as a whole are a necessary basis for investigating important public health issues [19]. Thus, gender- and age-specific evidence on behaviours at work and during leisure, preferably obtained using valid objective measurement methods, can feed informed initiatives promoting physical activity at and outside work [20–22].

Time spent in different behaviours, such as sitting, standing, and moving, within a specific time frame (e.g. all wake hours, or 100% of the working day), form parts of a whole; behaviours are “compositional” [23]. Compositional data are inherently constrained and multicollinear, meaning that they are not free to take any value, and that a change in time spent on one part of the composition will be accompanied by a change in time spent on at least one other part. During recent years compositional data analysis (CoDA [24]) has gained increasing attention within public and occupational health research [12, 23, 25–27] as a recommendable data processing and analysis approach that can effectively handle these challenges associated with compositional data. To date, no study has applied CoDA in an examination of the extent to which female and male office workers differ in the occurrence and temporal structure of objectively measured sedentary and physical activity behaviours during work and leisure.

## Aim

The aim of this study is to document compositions of sitting, standing and moving during working days for male and female office workers, and to determine the extent to which these compositions differ by gender, domain (work vs. leisure), and age.

## Method

### Participants

Objective measurements of sitting, standing and moving were collected in a convenience sample of five office sites within a large Swedish Government agency in the transportation sector (*n* = 119). The office sites were selected for the purpose of an intervention study addressing effects of implementing activity-based offices [28, 29]. In the present study, we used baseline data collected before any relocation. Inclusion criteria for workers into the study were current full-time employment and predominant office-based work. In addition, workers were required to have delivered accelerometer data on sitting, standing and moving (see below) on at least 1 day, including at least 8 h of valid recordings. Four workers were excluded due to insufficient accelerometry data, and an additional three workers due to lack of information about age. Thus, the study included a total of 112 workers, 55 men and 57 women. They worked in either cell offices (*n* = 64; 31 men and 33 women) or open-plan offices (*n* = 42; 21 men and 21 women); 6 had missing data on office type. All had access to sit-stand workstations. Necessary sample sizes were estimated for the original intervention study [28], and we considered the resulting study size to be sufficient to obtain estimates with a satisfying accuracy even of the effects of gender, domain (work vs leisure), and age on sitting, standing and moving. Ethical approval was obtained from the Regional Ethical Review Board in Uppsala, Sweden (Dnr.2015/118), and all workers provided their written informed consent prior to entering the study.

### Objective measurements of sitting, standing and moving

Sitting, standing and moving were monitored using a three-axial Actigraph GT3X-accelerometer (Actigraph LLC, Pensacola, FL, USA). The accelerometer, sampling data at 30 Hz, was worn on the worker’s right thigh for up to 8 consecutive days, including both workdays and days off work, 24 h/day to the extent possible [30, 31]. Recordings were analysed using the Acti4 software [30, 32], which for the purposes of the present study provided a time-line of behaviours in three categories, i.e. sitting, standing, and moving (walking, climbing stairs and running). Since we had access to recordings only from the thigh, we could not discriminate sitting from lying; but for the ease of reading we label this combined behaviour “sitting”. On basis of the behaviour time-lines, four exhaustive and mutually exclusive behaviours (“compositional parts”) were identified for work and leisure separately (as identified using diaries, see below); i.e. time spent sitting in bouts < 30 min; sitting in bouts ≥30 min; standing; and moving. The time-line of classified behaviours was imported to the Spike2 software (version 8; Cambridge Electronic Design, England) for visual inspection; periods with corrupted data were excluded, as well as non-wear time, defined as periods exceeding 4 h without any change in body position according to the accelerometer registration. Days were then included in further analyses if they contained at least 8 h of valid data. We chose this criterion to allow even for days with recordings shorter than 24 h; typically the first and last measurement day.

### Diaries

During the measurement days, workers completed diaries containing information on working hours, time in bed, and non-wear periods. On basis of the diaries we restricted further analyses to workdays, thus excluding, e.g. weekend days. Leisure was defined as awake time before and after work. Night-time sleep, identified by visual inspection of the objective recordings combined with information from the diaries, was excluded from further analysis.

### Questionnaires

Information about age (years), gender (man, woman), pain, weight and height was obtained from questionnaires filled in by the workers prior to the objective measurements. Pain intensity during the past week was assessed using a numeric rating scale, NRS 0–10 for various body regions, and categorized as no pain (NRS 0); mild pain (NRS 1–3); moderate pain (NRS 4–6); severe pain (NRS 7–10) [33]. Workers reporting pain in multiple body regions were classified according to the highest reported pain intensity in any region. Body Mass Index (BMI) was calculated as weight (kg) divided by height squared (cm^2^).

### Data analysis

SPSS software version 24 (IBM, USA) was used for descriptive statistical analysis, results presented as N (%) or mean (SD). CoDA was conducted using R version 1.1.3 (RStudio, Boston, MA, USA) [34]; specifically the “compositions” [35] and “robCompositions” packages [36].

#### Descriptive statistics

Daily time spent in each behaviour during work and leisure was averaged over all measured days for each worker, and expressed in minutes as well as percentages.

#### Log-ratio transformation of sedentary and physical activity behaviours

Individual compositions of behaviours were expressed for work and leisure separately in terms of isometric log-ratio (ilr) coordinates [24], using a sequential binary partition process [37] resulting in three ilr-coordinates expressing: A) sitting relative to non-sitting (i.e. standing and moving) behaviours; B) shorter (< 30 min) relative to longer (≥30 min) uninterrupted sitting bouts; C) standing relative to moving. We find that this set of ilr coordinates reflects behaviours in an intelligible and useful arrangement; and better than other alternatives for orthonormal partitions of the four compositional parts. The ilr-coordinates were defined as follows, based on eq. (2) in Dumuid et al. [37].

A: *sitting relative to non-sitting behaviours*
  $$ ilr\frac{sit}{non- sit}=\mathit{\ln}\left(\frac{\sqrt{sit<30\mathit{\min}\ast sit>30\mathit{\min}}}{\sqrt{stand\ast move}}\right) $$

B: *short (< 30 min) relative to long (≥30 min) sitting bouts*
  $$ ilr\frac{sit<30\mathit{\min}}{sit\ge 30\mathit{\min}}=\sqrt{\frac{1}{2}}\mathit{\ln}\left(\frac{sit<30\mathit{\min}}{sit\ge 30\mathit{\min}}\right) $$

C: *standing relative to moving*
  $$ ilr\frac{stand}{move}=\sqrt{\frac{1}{2}}\mathit{\ln}\left(\frac{stand}{move}\right) $$

Next, differences between men and women in ilrs A, B and C were examined using repeated-measures ANOVA with gender and age as between-subject factors and domain (work vs. leisure) as a within-subject factor. In addition to the main effects of gender, domain and age, the models included two-factor interaction terms between gender and age, gender and domain, and domain and age, as well as the three-factor interaction between gender, domain and age, which reflects the extent to which a possible effect of age on the difference between behaviours during work and leisure depends on gender. In these models, workers’ ages were centered on the mean age of the population to reduce collinearity. To examine the extent of data clustering within the five offices, we calculated Intra Class Correlations (ICCs) expressing variance between offices relative to total variance for all three ilrs in both work and leisure [38]. The three ICCs for work ranged between 0.00 and 0.01, and those for leisure between 0.00 and 0.05. Thus, we decided to not account for clustering in our statistical models, but rather interpret the eventual results with due consideration to the design effect of clustering (at the most 2.0; [39]) on the trustworthiness of estimated effect sizes. We did not include office type (i.e. cell vs. open-plan) in our statistical models, since an a-priori analysis showed a marginal association between office type and behaviours [28], and since the gender distribution was equal in the two office types, which disqualifies office type as a confounder of gender effects on behaviour. For a similar reason, we did not include the absolute duration (minutes) of time at work or in leisure. In keeping with the study aim of *documenting* physical behaviours in men and women of different age, rather than *explaining* these behaviours, we decided to not include pain and BMI In the models even though both may be associated with physical behaviour, and may differ between genders and according to age. Thus, the eventual results pertain to men and women ‘as they were’, and not to hypothetical workers with average pain and BMI. Partial eta squared (${\eta}_p^2$) and F-statistics were used as measures of effect size [40], and *p*-values were calculated as a measure of the likelihood of obtaining the observed effect if, in fact, the true effect is zero. Behaviours were illustrated using cumulative distribution plots in the standard, non-transformed space, and descriptive statistics of non-transformed as well as transformed data (ilr coordinates). In addition, transformed behaviours were illustrated using gender-stratified scatter plots portraying main effects and interactions of gender, domain and age.

## Results

### Participants and source data

Mean age of the 55 men and 57 women included in the study was 47.2 (range 26–65) and 45.4 (range 25–63) years, respectively (Table 1). Men had a mean BMI of 26.0 kg/m^2^ (SD 3.9) and women 24.5 kg/m^2^ (SD 4.6). Thirty-seven percent of the men and 52% of the women reported having had severe pain during the past week.

**Table 1:** Characteristics of the study population

	Men (***n*** = 55)		Women (***n*** = 57)	
	**N (%)**	**Mean (SD)**	**N (%)**	**Mean (SD)**
**Age**		47.2 (8.0)		45.4 (9.6)
**Weight (kg)**		85.8 (14.5)		70.5 (15.2)
**Height (m)**		1.82 (0.07)		1.69 (0.06)
**Body mass index (kg/m**^**2**^**)**		26.0 (3.9)		24.5 (4.6)
**Pain**				
**No pain**	14 (25.9)		11 (19.6)	
**Mild pain**	7 (13.0)		5 (8.9)	
**Moderate pain**	13 (24.1)		11 (19.6)	
**Severe pain**	20 (37.0)		29 (51.8)	

*Missing data men/women: Age 0/0; Weight 0/4; Height 0/0; Body Mass Index 0/4; Pain 1/1*

In total 468 working days with accelerometer data were available. Fifteen days were excluded due to less than 8 h of data, 14 of which were the first measurement day for a particular worker, leaving 453 days for further analysis. None of these days included non-wear periods. Thus, 4.0 complete days of accelerometer data were, on average, available from each worker (range 1–7 days), comprising 1917 min (SD 524) of work and 1805 min (SD 500) of leisure. According to the diaries, men and women worked, on average, 530 (SD 57) and 516 (SD 51) minutes per day, respectively, and they had 463 (SD 66) and 464 (SD 65) minutes of leisure (Table 2).

**Table 2:** Mean (SD between workers) minutes and percent time in Total and by behaviour (Sitting <30 min, Sitting ≥30 min, Standing, and Moving) for men and women, at work and during leisure; as well as ratios of behaviours prior to transformation into ilr coordinates

	Men (***n*** = 55)				Women (***n*** = 57)			
	Work		Leisure		Work		Leisure	
	Minutes	%time	Minutes	%time	Minutes	%time	Minutes	%time
**Total**	530 (57)	100	463 (66)	100	516 (51)	100	464 (65)	100
**Sitting < 30 min**	153 (59)	29.2 (9.9)	150 (36)	33.7 (8.2)	143 (41)	28.4 (7.6)	128 (37)	27.9 (6.3)
**Sitting ≥ 30 min**	230 (92)	42.5 (15.2)	129 (76)	26.8 (14.8)	203 (77)	38.4 (13.2)	124 (57)	27.1 (12.0)
**Standing**	110 (68)	21.4 (13.7)	125 (46)	26.8 (9.3)	132 (70)	25.8 (13.2)	153 (47)	32.4 (8.8)
**Moving**	37 (14)	6.9 (2.3)	59 (25)	12.7 (4.8)	38 (13)	7.4 (2.5)	59 (23)	12.6 (4.0)
**Sitting/Non-sitting**	3.44 (1.69)		1.88 (1.21)		2.89 (1.62)		1.49 (0.80)	
**Sitting < 30 min/Sitting ≥ 30 min**	0.88 (0.79)		1.97 (1.86)		0.86 (0.44)		1.47 (1.59)	
**Standing/Moving**	3.69 (3.71)		2.28 (0.87)		3.85 (2.62)		2.76 (1.08)	

### Behaviours expressed in standard space

**Fig. 1:**
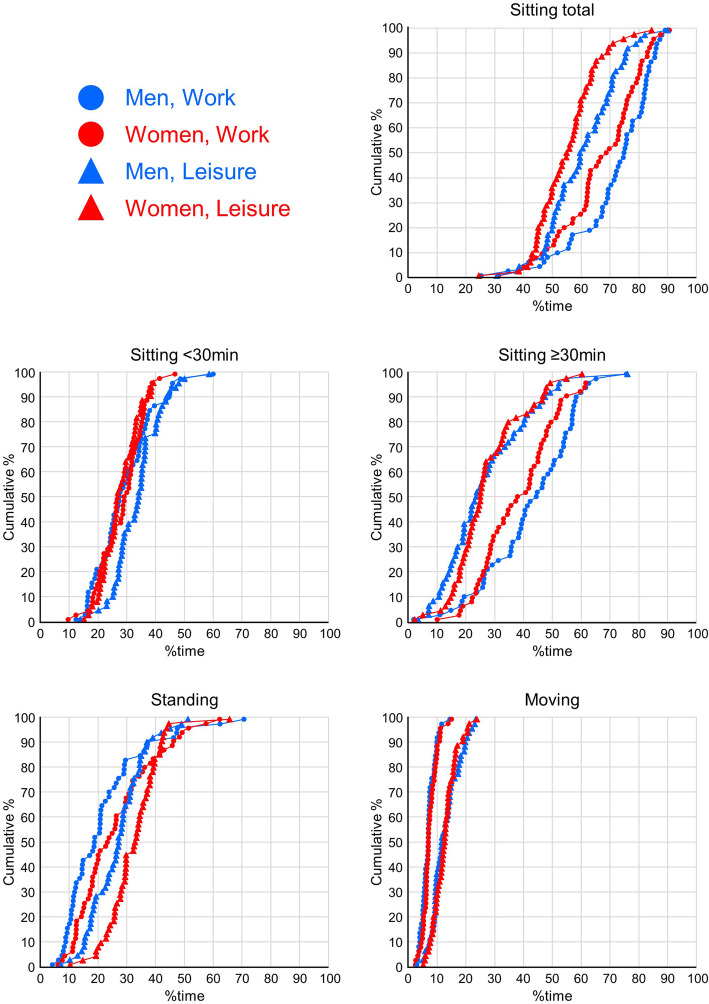
Cumulative distributions of percentages of time spent Sitting in total, Sitting in bouts < 30 min, Sitting in bouts ≥30 min, Standing, and Moving. Distributions are shown for men (blue symbols) and women (red symbols) at work (circles) and during leisure (triangles)

Distributions of percentage time spent Sitting in total, Sitting in bouts < 30 min, Sitting in bouts ≥30 min, Standing, and Moving are illustrated in Fig. 1, allowing the reader to examine central tendencies and dispersions in detail. Of note, all four behaviours occurred during both work and leisure for all participants. Fifty percent of the men and 34% of the women were sitting for more than 75% of their time at work (Fig. 1). During leisure, 13% of the men and 4% of the women were sitting for more than 75% of the time. At work, a larger proportion of time was spent in long sitting bouts than in short bouts, while percentages were more similar during leisure (Fig. 1, Table 2). Women had a higher proportion of time standing than men, both at work and during leisure. Time spent moving was rare for a major part of the workers, in particular at work (Fig. 1, Table 2). The dominating behaviour at work was sitting in bouts ≥30 min for both men and women (Fig. 1, Table 2); on average 230 min (43% of the total time at work) and 203 min (38%), respectively. Men spent 153 min (29% time) sitting in bouts < 30 min, 110 min standing, and 37 min moving at work (21 and 7%, respectively). The corresponding values for women were 143, 132, and 38 min (28, 26, and 7% time). During leisure, the dominating behaviour was short sitting bouts for men (150 min, 34% time; Table 2), while it was standing for women (153 min, 32%). Men and women spent 129 min (27%) and 124 min (27%) in sitting bouts ≥30 min, and both men and women spent 59 min moving (13% of the total time in leisure). In relative terms, sitting occurred considerably more than non-sitting for both genders, especially at work, and standing occupied a larger proportion of the non-sitting time than moving during work as well as leisure. This is expressed in the ratios of behaviours shown in Table 2. These ratios can be understood as the odds of finding a worker sitting (ratio Sitting/Non-sitting), finding a worker during a short bout while sitting (ratio Sitting< 30 min/Sitting≥30 min), and finding a worker standing during a non-sitting period of time (ratio Standing/Moving).

### Results based on ilr-transformed data (CoDA)

**Fig. 2:**
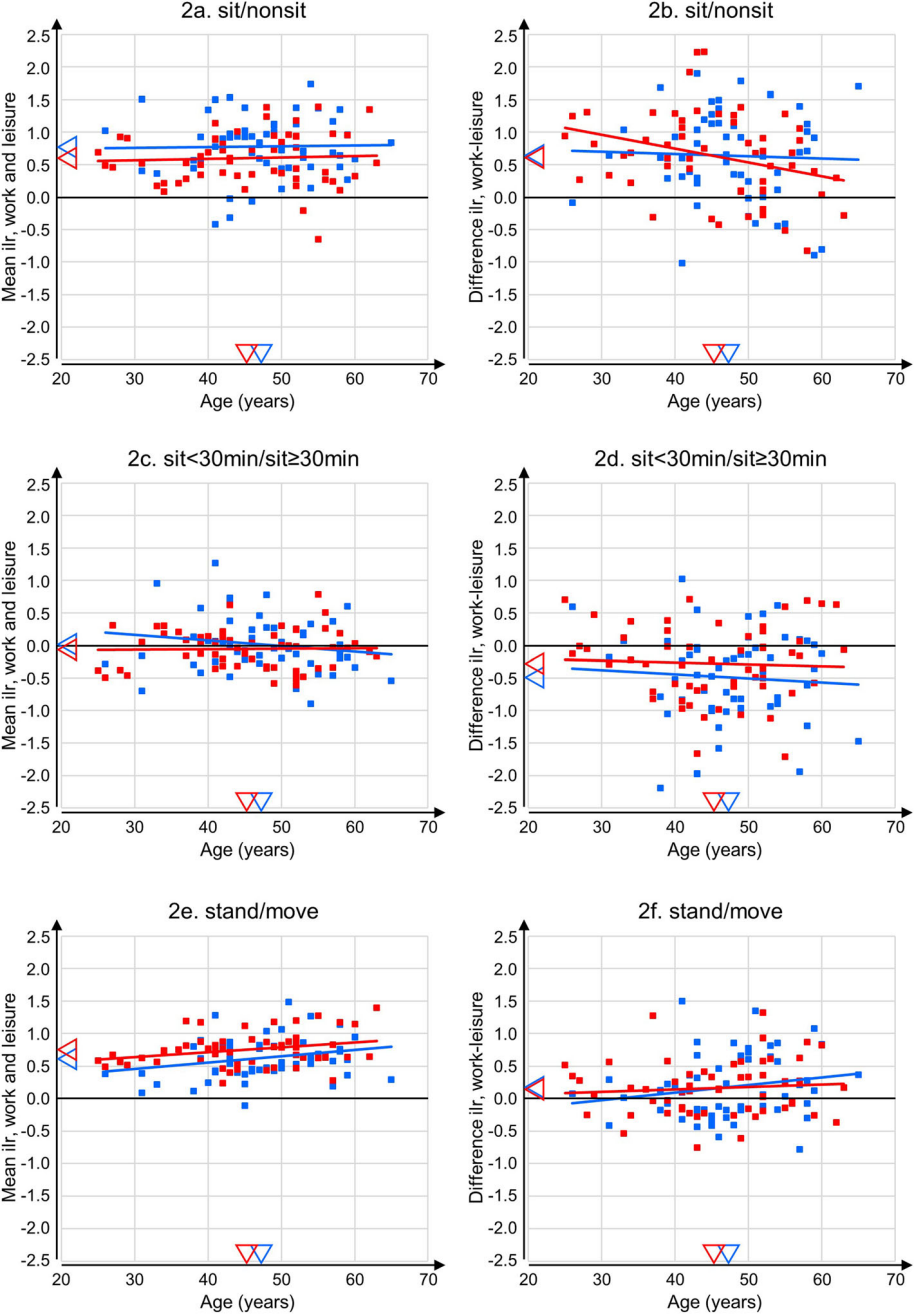
Gender-stratified scatter plots with regression lines, of age vs. the average ilrs of work and leisure (**a**, **c**, **e**), and of age vs. the difference between ilrs at work and during leisure (**b**, **d**, **f**). Blue and red symbols and lines illustrate data for men and women, respectively. Thus, **a**, **c**, **d** illustrate main effects of gender and age, as well as the gender*age interaction, while **b**, **d**, **f** illustrate the main effect of domain (i.e. the extent to which the work-leisure difference differs from 0); as well as the interactions between gender and domain; domain and age; and gender, domain and age. Triangles on the x-axes mark the mean age of men (blue) and women (red); triangles on the y-axes show the mean values of the dependent variable for men (blue) and women (red)

We found a main effect of gender on time spent sitting relative to non-sitting (${\eta}_p^2$ =0.04, *p* = 0.03; Table 3), and on standing relative to moving (${\eta}_p^2$ =0.07, *p* = 0.01). Men showed a higher ratio of sitting relative to non-sitting time than women (Fig. 2a, Table 2), and within the non-sitting time a lower ratio of standing relative to moving (Fig. 2e, Table 2). We also found that sitting time relative to non-sitting time was larger at work than during leisure (${\eta}_p^2$ =0.47, *p* < 0.01; Fig. 2b, Table 2), that within sitting, time in bouts < 30 min relative to bouts ≥30 min was larger during leisure than at work (${\eta}_p^2$ =0.26, *p* < 0.01; Fig. 2d, Table 2), and that standing occurred relatively more during the non-sitting time at work than it did in leisure (${\eta}_p^2$ =0.12, *p* < 0.01; Fig. 2f, Table 2). All interactions between gender and domain were very small (Table 3), indicating that differences in behaviours between work and leisure were similar for men and women (Table 2, Fig. 2 b, d, f). During non-sitting, time standing relative to moving changed with age (${\eta}_p^2$ =0.07, *p* = 0.01; Fig. 2e, Table 2); older workers spent a smaller proportion of their non-sitting time moving than younger workers. Age did not interact to any notable extent with gender for any of the three ilr (all ${\eta}_p^2$ 0.01 or less, *p* ≥ 0.25; Table 3, Fig. 2a, c, e), nor with domain (all ${\eta}_p^2$ =0.03 or less, *p* ≥ 0.09; Table 3, Fig. 2b, d, f). All of the three-factor interactions between gender, domain and age were small (all ${\eta}_p^2$ =0.01 or less, *p* ≥ 0.22; Table 3, Fig. 2 b, d, f).

**Table 3:** Effects (partial eta-squared (${\eta}_p^2$), F and P) of gender, domain (work vs. leisure), and age on time spent sitting, standing and moving, expressed in terms of ilr coordinates

	ilr sit/non-sit			ilr sit < 30/sit ≥ 30			ilr stand/move		
	${\eta}_p^2$	*F*	*P*	${\eta}_p^2$	*F*	*P*	${\eta}_p^2$	*F*	*P*
**Gender**	**0.04**	**4.64**	**0.03**	0.01	1.27	0.26	**0.07**	**7.49**	**0.01**
**Domain**	**0.47**	**97.50**	**< 0.01**	**0.26**	**38.23**	**< 0.01**	**0.12**	**14.52**	**< 0.01**
**Age**	< 0.01	0.13	0.72	0.01	0.94	0.34	**0.07**	**8.09**	**0.01**
**Gender·Domain**	< 0.01	0.06	0.81	0.02	2.69	0.10	< 0.01	< 0.01	0.96
**Gender·Age**	< 0.01	0.01	0.92	0.01	1.35	0.25	< 0.01	0.16	0.69
**Domain·Age**	0.03	2.86	0.09	< 0.01	0.41	0.53	0.02	2.32	0.13
**Gender·Domain·Age**	0.01	1.50	0.22	< 0.01	0.05	0.82	0.01	0.59	0.44

*The ANOVA models treated gender and age as between-subjects factors and domain as a within-subject factor*

## Discussion

This study of physical behaviours among office workers during working days documented compositions of sitting (or lying), standing and moving at work and during leisure among male and female workers of different age, and examined to which extent these compositions differed by gender and age.

In general, workers in the present population were sitting for extensive amounts of time, both at work and during leisure, and they moved only little. Compared with women, men spent proportionally more time sitting than non-sitting (Tables 2 and 3), and a larger part of their non-sitting time moving. For both genders, the proportion of sitting time in total, the occurrence of ‘short’ relative to ‘long’ sitting bouts, and the proportion of standing relative to moving during non-sitting time were all larger at work than during leisure. Older workers moved less during their non-sitting time than younger workers, while other behaviours changed only marginally with age. Differences in behaviour between work and leisure were similar for men and women and did not depend on age. These effects, with a possible exception of the gender difference in sitting relative to non-sitting, were marked (substantial values of ${\eta}_p^2$), and sufficiently confident (small *p*-values) to still be certain after taking into account the marginal effect of clustered data within offices.

A gender difference in the proportion of sitting relative to non-sitting time and in standing relative to moving is consistent with previous studies showing men in the general population to be, on average, more sedentary but, at the same time, more physically active than women [41, 42]. Similar gender differences have been found even in blue collars-workers [11, 27]. The present gender differences in compositions of behaviour may be explained by various factors both at and outside the office [43–45]. One hypothesis, based on previous studies, is that women and men with the same job title perform, to some extent, different work tasks, which may, in turn be associated with different patterns of behaviour at work [9, 43, 44, 46, 47]. During leisure, it is not uncommon that women perform a major part of the household chores such as cooking, cleaning, and doing the dishes and laundry [45], which are mainly performed standing. This likely contributes to the relatively lower ratio among women of sitting to non-sitting time, and the relatively higher ratio of standing to moving (Table 2). Some of the gender difference in behaviours may also be explained by the women in our population having pain to a larger extent than the men, and by the men having a somewhat larger BMI than the women (Table 1). Our results corroborate previous studies suggesting that women in general accumulate more time standing [12] and less time being physically active at moderate or higher intensities [41, 48] than men. Interestingly, however, we did not observe any notable interaction between gender and domain, meaning that differences in behaviours between work and leisure were similar for men and women. We did not, however, collect any additional information about the contexts at work and during leisure in which the behaviours were observed. Future studies exploring, comparing and explaining gendered behaviours would benefit from taking into consideration, e.g. work tasks, leisure time habits, physical capacity, and socioeconomic factors.

We found men to spend, on average, 72% and women 67% of their work time sitting, which is consistent with previous studies on office workers [49–51]. About 50% of the men, and 34% of the women in the present population spent more than 75% of their time at work sitting, predominantly in uninterrupted bouts longer than 30 min, which is also consistent with findings in other studies [51]. Moreover, the workers in the present population spent more than 50% of their leisure time sitting, corroborating previous evidence that office workers are, to a large extent, sedentary also outside work [50]. In total, a worker accumulated, on average, about 10 h of total sitting time per day. This agrees with some studies [49, 50] but is larger than in others [52]. The ratio of time spent sitting relative to non-sitting was smaller for both men and women during leisure than during work, suggesting that the workers compensated, to some extent, for their extensive sitting at work during non-work time. However, this increase in standing and moving during leisure is probably too small to combat the negative effects of extensive sitting at work [5]. Adults spending more than 10 h per day sedentary have been reported to have an almost 30% greater risk for premature death compared with those who spend less than 6 h per day sedentary [53], and total sedentary time has been strongly associated with negative effects on insulin resistance, which can contribute to type 2 diabetes and the metabolic syndrome [54]. Reallocating time from sedentary to physical activity has been estimated to decrease the risk for preterm death, with a greater risk reduction if sedentariness is replaced with physical activity at higher intensities [55]. A meta-analysis concluded that up to 60–75 min per day of physical activity at, at least, a moderate intensity, is needed to substantially reduce the risk for premature death associated with sedentary behaviour [5]. It is unlikely that workers in this study met up to these activity levels. The total time ‘moving’ was, indeed, about 90 min/day on average, but it included activities even at light intensities, such as slow walking. Standing also occurred to a considerable extent among the workers, but the increased muscle activity and metabolism associated with standing is probably not sufficient to lower the risks associated with extensive sedentariness [56]. Thus, even though breaking up sedentary time with physical activity at any intensity has been found to improve insulin [3], triglycerides, and glucose levels in the blood [57], intensities are probably not sufficient among the present workers to have any notable health effect. Thus, in the present population, bouts of more intense physical activity would likely be a priority [20, 58], in particular for those individuals with the largest proportions of time being sedentary. Gender differences in time spent sitting, standing and moving were small in the present population, and interventions aimed at reducing sitting time and promoting physical activity should target both men and women, with due consideration to gendered determinants of behaviour at work and during leisure.

We included age as a variable of interest since it has previously been found to be associated with sedentary and physical activity behaviours [15, 41]. Studies have reported age to be negatively associated with physical activity [17, 42], which is consistent with our findings of a higher ratio of standing relative to moving with increasing age. We did not, however, find any notable interactions between gender and age for the investigated behaviours, showing that changes in behaviours with age were similar for women and men. The observed decrease with age in time moving may be detrimental to health [5, 59], and men and women appear to be at risk to similar extents.

### Strengths and limitations

To our knowledge, this is the first study to report extensive data on compositions of sitting (including lying), standing and moving for both male and female office workers, during work as well as leisure, taking the workers’ age into account. Most studies of such behaviours have relied on self-reported data, which are likely less accurate [60–62]. Thus, a strength of the present study is the use of thigh-worn accelerometers to assess behaviours. Not only will these data likely be more accurate than self-reports [62], but they also allow for more detailed analyses, e.g. distinguishing between shorter and longer sitting bouts. The use of CoDA, which effectively addresses the inherent co-dependency of behaviours adding up to 100%time, is another major strength of the study.

We were not able to discriminate sitting from lying down. Thus ‘sitting’ is contaminated by time spent lying, i.e. the extent of literal sitting is overestimated. This overestimation may not be an issue of any major concern during working hours, while lying likely occurs to a greater extent during wake hours in leisure. Thus, in a large cohort study of Danish blue-collar workers, lying occurred, on average, for 6 min during an entire day at work, corresponding to just over 1% of the day, while participants were lying down for 54 min during leisure, i.e. just over 10% of an 8-h period [63]. While our inability to discriminate sitting from lying will result in biased data on (literal) sitting, we emphasize that both sitting and lying are physically inactive behaviours, and that our results regarding ‘sitting’ likely offer a valid proxy for the overall extent of inactive behaviours.

Another limitation is the lack of data on specific work tasks, which could have aided in interpreting and explaining the, to some extent, gendered patterns of behaviour. Thus, we recommend future studies to address this issue by including observations – quantitative as well as qualitative – of the context in which sitting, standing and moving occur. Only 26% of the included men and 20% of the women were free of pain. This pain prevalence was higher than what has been reported in most other studies [13]. That, together with the fact that the study rely on a sample of workers from only one organisation, suggests that the results should be generalized to other office populations only with caution.

## Conclusion

In conclusion, both men and women were, to a large extent, sitting (including lying) both at and outside work. Sitting time at work was predominantly spent in uninterrupted bouts longer than 30 min. Male workers spent proportionally more time sitting than non-sitting compared with female workers, and more of their non-sitting time moving. Older workers spent less of their non-sitting time moving than younger workers. Effect sizes for gender and age were, however, small and they did not depend on domain (work or leisure). In general, these office workers could likely benefit from interventions to reduce or further break up prolonged inactive periods, preferably by bouts of more intense physical activity. To this end, we recommend that interventions targeting excessive sedentary time and low physical activity among men and women should be based on gender-specific information on behaviours at work and during leisure, with an emphasis on the likely specific needs of older workers.

## Data Availability

The datasets generated and/or analysed during the current study are not publicly available due to confidentiality of some data sources, but processed data are available from the corresponding author on reasonable request.

## Abbreviation

CoDA: Compositional Data Analysis
